# Does bedtime matter among patients with chronic pain? A longitudinal comparison study

**DOI:** 10.1097/PR9.0000000000000747

**Published:** 2019-05-09

**Authors:** R. Kathryn McHugh, Robert R. Edwards, Edgar L. Ross, Robert N. Jamison

**Affiliations:** a Division of Alcohol and Drug Abuse, Department of Psychiatry, Harvard Medical School, McLean Hospital, Belmont, MA, USA; b Department of Anesthesiology, Pain Management Center, Harvard Medical School, Brigham and Women's Hospital, Boston, MA, USA

**Keywords:** Bedtime, Sleep, Chronic pain, Pain assessment, Pain app, Pain management

## Abstract

**Introduction:**

Chronic pain patients frequently report having sleep disturbances and many tend to stay up during the night and then sleep into the day.

**Objectives:**

This study was designed to compare a heterogeneous group of persons with chronic pain who reported typically going to bed between the hours of 9 pm and midnight with those who go to bed at other hours of the day and night.

**Methods:**

Two hundred seventy-nine participants were divided between those who reported going to bed between the hours of 9 pm and midnight (N = 205) and those who reported having atypical bedtimes (N = 74) based on pre–post questionnaire data and average pain assessments from a smartphone pain application (app).

**Results:**

Those individuals in the atypical bedtime group reported waking up more frequently and getting fewer hours sleep (*P* < 0.05). These individuals also reported significantly higher pain scores, activity interference, and taking more prescription opioid medication compared with those who had typical bedtimes (*P* < 0.05). Based on average 3-month daily assessments, those subjects with an atypical bedtime consistently reported more sleep disturbances, pain, activity interference, negative mood, and general worsening conditions over time, and elevated pain catastrophizing, pain-related disability, emotional distress scores, and more prescription medication for pain at 3-month follow-up (*P* < 0.01).

**Conclusion:**

These results support the importance of providers asking patients with pain about what time they typically go to bed at night to gain a greater understanding of their lifestyle habits. Future studies are needed to further determine the importance of maintaining a typical bedtime among patients with chronic pain.

## 1. Introduction

Chronic pain has a significant negative impact on sleep; people with chronic pain often report that they are sleep-deprived because they have trouble getting comfortable at night to get enough sleep.^15,20,24^ Individuals who have had chronic pain for many years frequently report that they tend to stay up during the night and then sleep into the day,^7^ and there can be a relationship between sleep–wake circadian rhythm misalignment and pain.^19^ An essential recommendation for healthy sleep is maintaining a regular bedtime and waketime.^14,16,17^

Although providers frequently document report of sleep disturbances among patients with chronic pain, few enquire about the habits (eg, time of day people sleep) of those who report difficulties with sleep. To the best of our knowledge, no study has examined the effect that maintaining a typical bedtime can have on how individuals manage chronic pain. This study was designed to compare a heterogeneous group of persons with chronic pain who report going to bed between the hours of 9 pm and midnight with those who go to bed at other hours of the day and night.

## 2. Methods

This study was approved by the internal review board of the Hospital. A smartphone pain app called “BWH PainApp” was developed, tested, and used in several clinical trials. The data used in this study were derived from control subjects with noncancer-related chronic pain from separate clinical trials conducted between January 2015 and December 2017; the study methods have been reported previously.^8,11–13^

As part of the initial evaluation collected on a smartphone pain app [eg, body mass index (BMI) and sleep hours], all participants were asked to respond to the same question “What time do you usually go to bed?” All subjects completed a packet of questionnaires at baseline and again after 3 months. All subjects received push notification on their smartphone at a convenient daily time of their choosing and were encouraged to complete a 5-item daily assessment on the pain app consisting of ratings of sleep disturbances, pain intensity, activity interference, mood disturbance, and how much things had changed over the past 24 hours on a visual analog scale (1–10 where 1 = better, 5 = same, and 10 = worse). Each participant received $25 after completing the baseline questionnaires and $50 after completing the 3-month assessments.

### 2.1. Participants

We recruited individuals with chronic pain to participate in the studies.^8,11–13^ All participants needed to be 18 years or older and own a study-compatible smartphone (iPhone or Android device). Other inclusion criteria included (1) having chronic pain for >3 months' duration, (2) averaging 4 or greater on a pain intensity scale of 0 to 10, and (3) able to speak and understand English. Subjects were excluded from the study if they had (1) any cognitive impairment that would prevent them from completing procedures, (2) any clinically unstable medical condition, (3) a pain condition requiring urgent surgery, (4) a present psychiatric or substance use disorder, and (5) visual impairment or motor impairment that would interfere with use of a smartphone. All subjects identified their area of primary pain using a body map.

### 2.2. Measures

The study measures were completed at the time of recruitment and 3-month follow-up questionnaires. They included the Brief Pain Inventory,^4,5^ the Pain Catastrophizing Scale,^21,22^ the Pain Disability Index,^23^ and the Hospital Anxiety and Depression Scale.^2,26^

### 2.3. Statistical analyses

Subjects were divided into 2 groups based on their reported typical bedtimes: (1) typical bedtimes (9 pm to 12 midnight, based a review of the literature of optimal sleeping times^1,3,6,18,19^) and (2) atypical bed times (12:01 am–8:59 pm). Significant differences between groups at baseline and follow-up were assessed (IBM, SPSS v.25). The primary variables of interest were group differences of activity interference, disability, catastrophizing, and mood with a cutoff alpha level of *P* < 0.05. We also controlled for those descriptive factors that correlated with outcome using linear regression analyses and including those variables that significantly differentiated the groups in the model with Bonferroni analyses to adjust for multiple comparisons when appropriate.

## 3. Results

Two hundred seventy-nine (N = 279) participants who were followed for 3 months were included. Average age of the participants was 51.0 ± 13.9 (range 18–92), 74.9% were women, and 85.6% were Anglo-American. Thirty-four percent had primary low back pain, and they averaged 11.5 ± 11.4 years of pain. Their average BMI was 30.4 ± 7.4, and the average number of daily entries was 57.8 ± 59.5 (range 1–90). Forty-six percent was prescribed opioids, 18.6% anticonvulsants, 14.2% muscle relaxers, 8.2% benzodiazepines, and 8.2% antidepressants.

Two hundred five (N = 205, 73.5%) of the subjects reported routinely going to bed between the hours of 9 pm and midnight (9 pm = 22; 10 pm = 72; 11 pm = 80; and 12 am = 31). Of 74 subjects who had atypical bedtimes, 34 (12.2%) subjects reported going to bed after midnight and before 5 am, while 40 (14.4%) reported typically going to bed after 5 am

Table 1 presents differences between the typical bedtime and atypical bedtime groups at baseline. Women, those with lower BMI scores, and those who reported more sleep hours significantly more often reported keeping a typical bedtime, but these factors did not consistently affect the findings when controlling for these variables using linear regression analyses. Those individuals in the atypical bedtime group reported waking up more frequently and getting fewer hours sleep (*P* < 0.05). These individuals also reported significantly higher pain scores and more activity interference. Those typical bedtime subjects who reported going to bed between 9 pm and midnight every evening reported taking more over-the-counter medication (46.8% vs 32.4%, χ^2^ = 4.6; *P* < 0.05), but less often taking prescription opioid medication for their pain compared with those who reported an atypical bedtime (37.2% vs 65.6%, χ^2^ = 14.8, *P* < 0.001), In general, those who maintained atypical bedtimes reported taking other prescription medications more often, but no significant differences were found between groups on any of the other categories of medications.

**Table 1:**
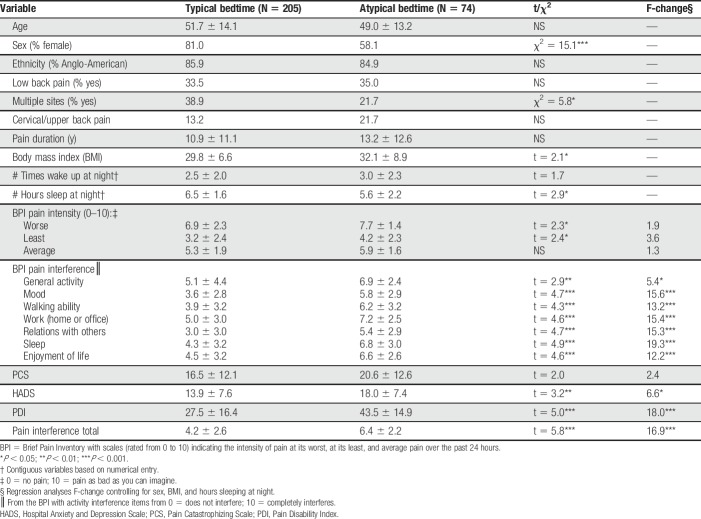
Descriptive characteristics and baseline information of patients with pain who reported typically going to bed (1) between 9 pm and midnight (N = 205), or (2) at odd hours after midnight (N = 74).

Table 2 demonstrates that those subjects with an atypical bedtime consistently reported more sleep disturbances, increased pain, more activity interference and negative mood, and general worsening conditions based on 3-month daily assessments (*P* < 0.01). Three-month follow-up questionnaire scores identified consistently elevated scores on the Pain Catastrophizing Scale, Hospital Anxiety and Depression Scale, Pain Disability Index, and mean pain interference (*P* < 0.01).

**Table 2:**
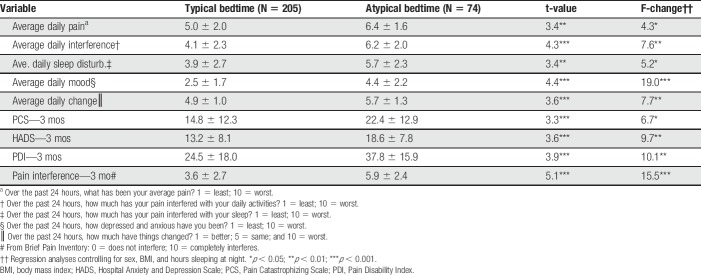
Differences between groups on daily assessments (pain, activity interference, sleep disturbances, mood, and whether things have gotten better or worse) and standardized questionnaires after 3 months.

## 4. Discussion

This secondary-data-analysis study was designed to examine differences between persons with chronic pain who report going to bed between 9 pm and midnight compared with those who reported atypical bedtimes of going to bed after midnight and during daytime hours. Our main findings showed that patients who reported going to bed at atypical hours tended to be doing less well overall even controlling for sex, BMI, and sleep duration. They report greater pain, decreased activity levels, more anxiety and depression, and a greater tendency to catastrophize at baseline and after 3 months. These results imply that enquiring about when patients with chronic pain typically go to bed each night might help identify those individuals who are having the greatest difficulty in managing their pain.

Patients with pain who have a significant mood disorder, catastrophize, and are more disabled due to their pain tend to ruminate about pain and may tend to focus on symptoms that can add to problems with sleep. There is evidence that patients who demonstrate the most emotional distress are prone to be prescribed medication for pain and sleep.^9^ However, those with greater negative affect tend not to benefit from opioids and other medications for pain.^10,25^ The results of this study suggest that despite relying more on prescription medication, including prescription opioids for pain, those with poor bedtime habits tended to do less well overall compared with those who take less medication and maintain a more typical bedtime.

There is evidence that cognitive-behavioral therapy and information about improving sleep can be beneficial for persons with chronic pain.^7,14^ Among the recommendations is to stick to a regular normal bedtime schedule. The results of this study suggest that those individuals who acquire a habit of getting into bed in the early morning hours are prone to being more depressed and socially isolated. Additional studies are needed to determine whether maintaining a more typical bedtime would improve functioning in people with chronic pain.

There are several limitations of this study. First, these data are correlational, and no causal relationships can be implied. There may be many reasons for individuals to go to bed at atypical hours unrelated to their condition. Second, the data were based on self-report. This study would be strengthened with the use of activity monitors, and future trials may consider including more objective ways to track bedtime and sleep quality. The subjects were also asked to identify typical bedtimes only on one occasion, and it is uncertain whether this changed with time. Also, we did not determine whether the participants had a previous sleep disorder history or were employed working nights. Despite these limitations, the results of this study support the importance of providers asking persons with chronic pain about what time they typically go to bed at night to gain a greater understanding of their lifestyle habits. Future studies are needed to further determine the importance of a typical bedtime on quality of life among patients with pain.

## Disclosures

The authors have no conflicts of interest to declare.

Funding for this study was made available through a pilot grant from the Mayday Fund and a government grant through the Center for Future Technologies in Cancer Care (through Boston University #015403-03). Investigator-initiated grants were also obtained from Brownmed, Inc, and Neurometrix, Inc.
